# Identification, function, and application of 3-ketosteroid Δ1-dehydrogenase isozymes in *Mycobacterium neoaurum* DSM 1381 for the production of steroidic synthons

**DOI:** 10.1186/s12934-018-0916-9

**Published:** 2018-05-18

**Authors:** Ruijie Zhang, Xiangcen Liu, Yushi Wang, Yuchang Han, Junsong Sun, Jiping Shi, Baoguo Zhang

**Affiliations:** 1 Lab of Biorefinery, Shanghai Advanced Research Institute, Chinese Academy of Sciences, No. 99 Haike Road, Pudong, 201210 Shanghai China; 2 School of Life Science and Technology, ShanghaiTech University, Shanghai, 201210 China; 3 Shanghai Institute of Biochemistry and Cell Biology, Chinese Academy of Sciences, Shanghai, 200031 China; 4 University of Chinese Academy of Sciences, Beijing, 100049 China

**Keywords:** *Mycobacterium*, 3-Ketosteroid-∆1-dehydrogenase, 22-Hydroxy-23,24-bisnorchol-4-ene-3-one (4HP), 1,4-Androstadiene-3,17-dione (ADD) steroids

## Abstract

**Background:**

3-Ketosteroid-Δ1-dehydrogenase (KstD) is a key enzyme in the metabolic pathway for chemical modifications of steroid hormones. Only a few KstDs have thus far been characterized biochemically and applied for the production of steroidal pharmaceutical intermediates. Three KstDs, KstD1, KstD2, and KstD3, were identified in *Mycobacterium neoaurum* DSM 1381, and they shared up to 99, 85 and 97% amino acid identity with previously reported KstDs, respectively. In this paper, KstDs from *M. neoaurum* DSM 1381 were investigated and exemplified their potential application for industrial steroid transformation.

**Results:**

The recombinant KstD2 from *Bacillus subtilis* exhibited higher enzymatic activity when 4-androstene-3,17-dione (AD) and 22-hydroxy-23, 24-bisnorchol-4-ene-3-one (4HP) were used as the substrates, and resulted in specific activities of 22.40 and 19.19 U mg^−1^, respectively. However, the specific activities of recombinant KstD2 from *Escherichia coli,* recombinant KstD1 from *B. subtilis* and *E. coli*, and recombinant KstD3, also fed with AD and 4HP, had significantly lower specific activities. We achieved up to 99% bioconversion rate of 1,4-androstadiene-3,17-dione (ADD) from 8 g L^−1^ AD after 15 h of fermentation using *E. coli* transformant BL21-*kstD2*. And in vivo transcriptional analysis revealed that the expression of *kstD1* in *M. neoaurum* DSM 1381 increased by 60.5-fold with phytosterols as the substrate, while the mRNA levels of *kstD2* and *kstD3* were bearly affected by the phytosterols. Therefore, we attempted to create a 4HP producing strain without *kstD1*, which could covert 20 g L^−1^ phytosterols to 14.18 g L^−1^ 4HP.

**Conclusions:**

In vitro assay employing the recombinant enzymes revealed that KstD2 was the most promising candidate for biocatalysis in biotransformation of AD. However, in vivo analysis showed that the cellular regulation of *kstD1* was much more active than those of the other *kstDs* in response to the presence of phytosterols. Based on the findings above, we successfully constructed *E. coli* transformant BL21-*kstD2* for ADD production from AD and *M. neoaurum* DSM 1381 Δ*kstD1* strain for 4HP production using phytosterols as the substrate.

**Electronic supplementary material:**

The online version of this article (10.1186/s12934-018-0916-9) contains supplementary material, which is available to authorized users.

## Background

Many actinobacteria, including *Mycobacterium*, *Streptomyces,* and *Rhodococcus*, can utilize natural sterols as carbon and energy sources [1–3], and interruption of those organisms’ unique catabolic pathways often led to the accumulation of steroid hormone derivatives [4, 5], some of which are important precursors, such as C19-steroids (4-androstene-3,17-dione [AD], 1,4-androstadiene-3,17-dione [ADD], and 9α-hydroxy-4-androsten-3,17-dione [9-OHAD]), for the production of steroidal drugs [6–8]. Phytosterols are found in plant seeds and can be utilized for the production of AD, ADD, and 9-OHAD using *Mycobacterium* sp. NRRL 3805B [9], *Mycobacterium* sp. NRRL 3683B [1], and *Mycobacterium neoaurum* NwIB-yV [10], respectively. However, besides the low conversion rate [11], another major drawback of microbial transformation of phytosterols is the concomitant accumulation of byproducts due to the excessive enzymatic bioprocessing of the industrial strains [12]. Although actinobacteria strains were reported to be able to perform chemical modifications on C22-steroids, such as 22-hydroxy-23, 24-bisnorchola-4-en-3-one (4HP), 22-hydroxy-23, 24-bisnorchola-1,4-dien-3-one (HPD) and 9,22-dihydroxy-23,24-bisnorchol-4-ene-3-one (9-OHHP) [1, 12, 13] which are all valuable precursors for the synthesis of progestational and adrenocortical hormones, more informations in detailed mechanisms are urgently needed for the full industrial application.

**Fig. 1:**
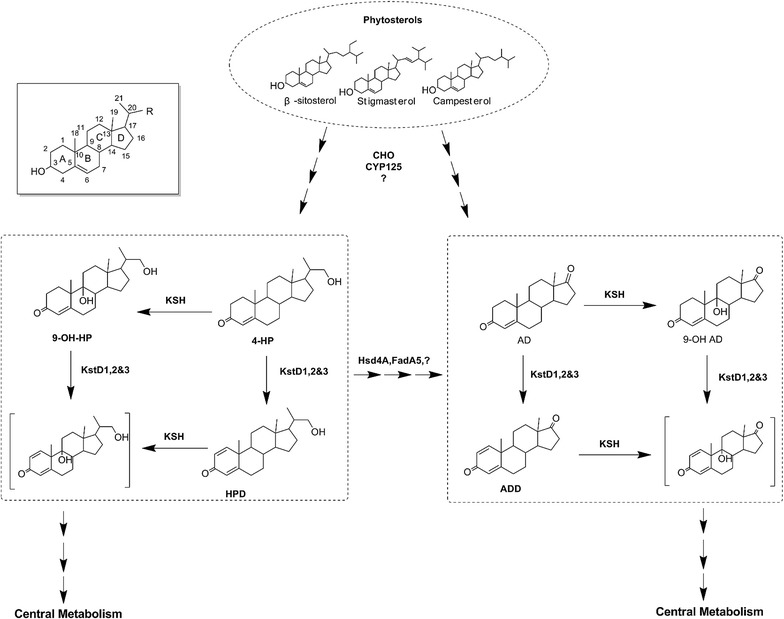
An overview of proposed pathway for phytosterols degradation in mycobacteria. Phytosterols can be transformed to vary valuable intermediates, including C19-steroids (4-androstene-3,17-dione [AD], 1,4-androstadiene-3,17-dione [ADD], and 9α-hydroxy-4-androsten-3,17-dione [9-OHAD]) and C22-steroids (22-hydroxy-23, 24-bisnorchola-4-en-3-one [4HP], 22-hydroxy-23, 24-bisnorchola-1,4-dien-3-one [HPD] and 9,22-dihydroxy-23,24-bisnorchol-4-ene-3-one [9-OHHP]). KstD, 3-ketosteroid-Δ1-dehydrogenase; KSH, 3-ketosteroid-9α-hydroxylases; CYP125, cytochrome P450 125; CHO, cholesterol oxidase; Hsd4A, 17β-hydroxysteroid dehydrogenase and β-hydroxyacyl-CoA dehydrogenase; FadA5, thiolase FadA5

Earlier research has, to some extent, clarified the sterol metabolic pathways in the actinobacteria based on the identification of intermediates [2]. Generally, 3-oxidation and the partial or full removal of aliphatic chains at C17 of the sterols, a process similar to fatty acid β-oxidation, are initial steps for the degradation of sterols, leading to the synthesis of 3-keto compounds such as 4HP and AD (Fig. 1) [2, 14]. Enzymes, including cholesterol oxidase (CHO), 17β-hydroxysteroid dehydrogenase/β-hydroxyacyl-CoA dehydrogenase (Hsd4A), thiolase FadA5 (FadA5), and cytochrome P450 125 (CYP125), have been reported to be involved in that degradation of sterols [12, 15]. After the degradation, as shown in Fig. 1, 4HP and AD can then be converted to HPD and ADD, respectively, by 3-ketosteroid-Δ1-dehydrogenase (KstD) [10, 12]. And HPD or ADD enter downstream oxidative process in cells after the 9α-hydroxylation, which is catalysed by 3-ketosteroid-9α-hydroxylases (KSH) [16, 17].

KstD removes hydrogen atoms of the C-1 and C-2 in the A-ring of the polycyclic ring structure of 3-ketosteroids in substrates including AD, hydrocortisone acetate, and 9-OHAD (Fig. 1) [12, 18, 19]. Although recent studies also focused on the genetic removal of KstD from native cells [10], the genetic manipulation in some strains might be challenging since host cells could contain multiple *kstDs* with each gene playing a different role in engineering the bacteria and altering its metabolic pathway [10, 13]. For example, in *M. neoaurum* ATCC 25795, KstD3 and KstD1 contributed to the conversion of AD and 9-OHAD, respectively, whereas KstD2 was probably involved in Δ1- dehydrogenation of C22 intermediates [10, 12]. Besides, enormous attempts have been made to express these KstDs heterologously in *Escherichia coli*, *Bacillus subtilis*, *Pichia pastoris,* and *Corynebacterium crenatum* to accomplish biotransformation of sterols [20–24]. For example, an *E. coli* transformant yielded 5.6 g L^−1^ ADD during fed-batch fermentation [23]. A *B. subtilis,* expressing codon-optimized *kstD* gene from *M. neoaurum* JC-12, produced 8.76 g L^−1^ ADD by whole-cell biocatalysis [24]. Prominently, a KstD overexpressing *C. crenatum* can transform 83.87% of the AD to ADD [22].

It has been reported that *Mycobacterium neoaurum* DSM 1381 (*Mycobacterium parafortuitum complex* MCI 0617) was able to transform phytosterols to 4HP and HPD, and the molar ratio of HPD/4HP reached 16.61:1, suggesting that *M. neoaurum* DSM 1381 owned KstDs with high catalytic activities [11]. In this study, transcriptional analysis and heterologous overexpression of KstDs were performed to determine the isoenzymes’ biochemical roles in the biotransformation of phytosterols to HPD. KstD2 can be used to construct recombinant strains that could efficiently transform AD to ADD, and *ΔkstD1* mutant of *M. neoaurum* DSM 1381 was found to synthesize 4HP, all of which could bring a substantive impact on the current pharmaceutical industry.

## Methods

### Bacterial strains, plasmids, and reagents

*Mycobacterium neoaurum* DSM 1381 was purchased from Deutsche Sammlung von Mikroorganismen und Zellkulturen (DSMZ), and the MP01 medium used to maintain *M. neoaurum* DSM 1381 at 30 °C was (g L^−1^): corn steep powder 10.0, glucose 6.0, K2HPO4 · 3H_2_O 2.0, MgSO_4_ · 7H_2_O 1.0, NaNO_3_ 2.0 and Tween 80 1.0 mL (v/v) (adjusted to pH 7.5). 5 g L^−1^ phytosterols were added to MP01 medium to measure the steroid bioconversion performances of the *M. neoaurum* DSM 1381 and related transformants. For high steroid concentration fermentation, phytosterols were prepared in (2-Hydroxypropyl)-β-cyclodextrin (HP-β-CD) (1:1.5). *E. coli* DH5α, *E. coli* BL21 (DE3) and *B. subtili*s 6051a were cultivated with Luria–Bertani medium (LB medium) at 37 °C and 200 rpm for molecular cloning and heterologous expression of *kstD* genes. All the other strains and plasmids were listed in Table 1. Oligonucleotides are listed in Additional file 1: Table S1. All the plasmids were constructed with ClonExpress^®^ II One Step Cloning Kit (Vazyme Biotech Co.Ltd. Nanjing, China). Restriction enzymes and other molecular biology reagents were purchased from Thermo Fisher Scientific.

**Table 1:** Bacterial strains and plasmids used in this study

Name	Description	Source/references
Strains		
*M. neoaurum* DSM 1381	Mutant of *M. neoaurum* ATCC 25790 which can accumulate HPD, 4HP and ADD	[11]
Δ*kstD1*	Mutant of *M. neoaurum* DSM 1381 deleted *kstD1*	This study
HK1/HK2/HK3	Δ*kstD1* harboring pMV306hsp-*kstD1*/pMV306hsp-*kstD2*/pMV306hsp-*kstD3*	This study
PK1/PK2/PK3	Δ*kstD1* harboring pMV306Pk1-*kstD1*/pMV306Pk2-*kstD2*/pMV306Pk3-*kstD3*	This study
BL21-pET-28a(+)	*E. coli* BL21 (DE3) harboring empty plasmid pET-28a(+)	This study
BL21-*kstD1*/BL21-*kstD2*/BL21-*kstD3*	*E. coli* BL21 (DE3) harboring plasmid pET28a-*kstD1*/pET28a-*kstD2*/pET28a-*kstD3*	This study
6051a-pHT01	*B. subtilis* 6051a harboring empty plasmid pHT01	This study
6051a-*kstD1*/6051a-*kstD2*/6051a-*kstD3*	*B. subtilis* 6051a harboring plasmid pHT01-*kstD1*/pHT01-*kstD2*/pHT01-*kstD3*	This study
Plasmids		
PJV53	pLAM12 carrying Che9c 60–61 under control of the acetamidase promoter	[38]
pGOAL19	Source of hygromycin (Hyg) cassette	[37]
pET-28a (+)	*E. coli* expression vector, Kan^R^	Novagen
pHT01	*B. subtilis* expression vector pHT01; containing Pgrac promoter; Amp^R^ for *E. coli*; Cm^R^ for *B. subtilis*	[34]
pMV306	Mycobacterium integrative vector with single copy, without *hsp60* promoter, Kan^R^	[39]
pMV306hsp	pMV306 contains *hsp60* promoter	[40]
pET24a-K1UHD	pET-24a(+) contains upstream and downstream of *kstD1* gene flanking *Hyg* cassette	This study
pHT01-*kstD1*/pHT01-*kstD2*/pHT01-*kstD3*	pHT01 possessing *kstD1* or *kstD2* or *kstD3* gene	This study
pET28a-*kstD1*/pET28a-*kstD2*/pET28a-*kstD3*	pET-28a (+) possessing *kstD1* or *kstD2* or *kstD3* gene	This study
pMV306hsp-*kstD1*/pMV306hsp-*kstD2*/pMV306hsp-*kstD3*	pMV306hsp possessing *kstD1* or *kstD2* or *kstD3* gene	This study
pMV306Pk1- *kstD1*	pMV306 carrying the 441 bp upstream region together with *kstD1* gene	This study
pMV306Pk2-*kstD2*	pMV306 carrying the 726 bp upstream region together with *kstD2* gene	This study
pMV306Pk3- *kstD3*	pMV306 carrying the 1347 bp upstream region together with *kstD3* gene	This study

Phytosterols were purchased Jiangsu Yuehong Group Company (Jiangsu, China). AD and ADD were obtained from Sigma. 4HP was from Steraloids (Newport, RI, USA). Phenazine methosulphate (PMS) and 2,6-dichlorophenolindophenol (DCPIP) were obtained from Sigma-Aldrich (Shanghai, China). Isopropyl β-d-1-thiogalactopyranoside (IPTG), Ampicillin 100 µg mL^−1^, kanamycin 50 µg mL^−1^, *Hyg*romycin 150 µg mL^−1^ or 10 µg mL^−1^ chloramphenicol was supplemented into medium when needed.

### Bioinformatic analysis

The genome of *M. neoaurum* DSM 1381 was isolated and cut with Covaris M220 to fragments of 400–500 bp length, and libraries of 500 bp genomic DNA fragments were builded and sequenced using Illumina Miseq (Majorbio, Shanghai). Then GS De Novo Assembler v2.8 were employed to perform genome assembly. And the genes were predicted using Glimmer 3.02 (http://www.cbcb.umd.edu/software/glimmer/) and annotated with BLAST 2.2.25 + . The putative genes for KstD were identified by comparing with the known KstD protein sequences taken from the NCBI database. Then the amino acid (aa) sequences of the identified KstDs in *Mycobacterium neoaurum* ATCC 25795 [10], *Mycobacterium neoaurum* NwIB-01 [18], *Mycobacterium* sp. VKM Ac-1817D [25], *Mycobacterium* sp. VKM Ac-1816D [25], *Mycobacterium smegmatis* mc^2^155 [13], *Rhodococcus rhodochrous* DSM43269 [26], *Rhodococcus ruber* strain Chol-4 [27, 28], *Rhodococcus erythropolis* SQ1 [29–31] were used to construct phylogenetic tree using the MEGA6 software with ClustalW and neighbor-joining algorithm. FgenesB was used to predict Operons and ORFs closed to the three *kstDs* (http://linux1.softberry.com/berry.phtml?topic=fgenesb&group=programs&subgroup=gfindb). The putative binding sites of transcription factors KstR [32] and KstR2 [33] were searched among the regions 500 bp upstream plus ORFs or operon of *kstDs* by software package UGENE 1.27.0. The positional weight matrices (PWM) were built from the known KstR operator motifs of mycobacteria and the sites with quality parameter score no less than 85% were used for further analysis.

### Construction of recombinant BL21-*kstD1*/BL21-*kstD2*/BL21-*kstD3* and 6051a-*kstD1*/6051a-*kstD2*/6051a-*kstD3* strains

The *kstD* genes were amplified from the *M. neoaurum* DSM 1381 genome DNA with the corresponding primers by PCR. The PCR products were cloned into BamHI/HindIII-digested *E. coli* expression vector pET-28a (+) (Novagen) or BamHI/SmaI-digested *B. subtilis* expression vector pHT01 [34], all expression plasmids were purified from *E. coli* DH5α and verified by DNA sequencing. pET28a-*kstD1*/pET28a-*kstD2*/pET28a-*kstD3* were transformed into *E. coli* BL21 (DE3) following standard protocols; pHT01-*kstD1/*pHT01-*kstD2/*pHT01-*kstD3* were transformed into *B. subtilis* 6051a by the method described previously [35]. The positive transformants of the recombinant *E. coli* BL21 (DE3) strains BL21-*kstD1/*BL21-*kstD2/*BL21-*kstD3* and *B. subtilis* 6051a strains 6051a-*kstD1/*6051a-*kstD2/*6051a-*kstD3* were selected with the supplement of kanamycin and chloramphenicol in the LB agar plates, respectively and then verified by DNA sequencing. The expression of *kstDs* in *E. coli* BL21 (DE3) and *B. subtilis* 6051a were checked by SDS-PAGE, KstD enzymatic assay and whole-cell biotransformation of 4HP and AD.

### Whole-cell steroid biotransformation with *E. coli* BL21 (DE3) and *B. subtilis* 6051a recombinants

The recombinant strains for heterologous expression of *kstD* genes were cultured in LB medium for 8 h at 37 °C. Then the cells inoculated in LB medium (30 mL per 250 mL flask) with 10% inoculum size. The LB mediums were previously supplied with 1 mM IPTG and 1 g L^−1^ AD or 4HP which was dissolved in HP-β-CD (final concentration, 0.7%) and Tween 80 (final concentration, 0.1%). The cultures were incubated at 37 °C and 200 rpm for 12 h and then sampled to detect the substrate conversion rates using HPLC. Transformation capacity of BL21-*kstD2* on AD was tested in the fermentation processing mentioned above except the medium changed to Terrific Broth medium which is richer in nutrition. 8 g L^−1^ AD, HP-β-CD (1:7) and Tween 80 (final concentration, 0.1%) were added in Terrific Broth medium.

### Expression analysis by RT–qPCR

Mid-log exponential phase cultures of *M. neoaurum* DSM 1381 and Δ*kstD1* (fermentation time: 33 h-36 h) on MP01 medium added with 5 g L^−1^ phytosterols and MP01 medium were collected and used to extract total RNA. After wall-breaking with liquid nitrogen grinding, standard protocol for RNAiso Plus reagents was followed to isolate RNA. Recombinant DNase I (TAKARA) was employed to eliminate the contaminating genomic DNA. The quality and concentration of RNA were evaluated by agarose gel electrophoresis, PCR test and Nano Drop 2000 (Thermo Scientific). Besides, the trace residual genomic DNA in the total RNA was removed during the reverse transcription using PrimeScript™RT reagent Kit with gDNA Eraser. 0.8 μg total RNA with gDNA Eraser and its buffer was first incubated at 42 °C, and then PrimeScript RT Enzyme Mix I, RT Primer Mix, 5 × PrimeScript Buffer 2 (for Real Time) were added to 20 mL and reacted at 37 °C for 15 min; 85 °C for 5 s; 4 °C. The cDNA products were diluted and analyzed on ViiA™ 7 Real-Time PCR System (Applied Biosystems^®^). SYBR^®^ Premix Ex Taq™ (Tli RNaseH Plus) and primers (16SrRNAf&r, RTkd1f&r, RTkd2f&r, RTkd3f&r) were included in the reaction mixture of RT-qPCR and all reaction mixtures were prepared in triplicate. The reaction program was as follow: 95 °C for 15 min; 40 cycles of 95 °C for 10 s; 60 °C for 40 s; Melting Curve Stage was from 60 to 95 °C. The relative fold change of each gene was calculated using the 2^−∆∆Ct^ algorithm and 16S rRNA was used as a reference gene. All the RT-qPCR experiments repeated for three times. The MP01 medium was set as native control. The fold change in the Fig. 5 indicates the increase degree of the expression level of the *kstDs* after accounting for the level detected in the MP01 medium.

### Deletion of *kstD1* gene

Gene deletion was performed by the methods described by Alessandro Cascioferro et al. [36]. A 1.6-kb hygromycin (*hyg*) cassette was amplified from PGOAL19 [37] using the primers hyg*dif*-f&r, which contain the reported *dif* sequence of *M. smegmatis*, and then the cassette was subcloned in the EcoRI site of the plasmid pET-24a (+), then the upstream and downstream of *kstD1* were ligated to the sides of the excisable cassette via HindIII and XbaI restriction sites, respectively. The result pET24a-K1UHD plasmid was the template to amplify the recombineering DNA fragments using primers kd1Uf and kd1Dr. The PCR products were transformed into *M. neoaurum* DSM 1381 harboring pJV53 which was employed to increase recombination efficiency [38]. The successful recombination colonies were selected using both kanamycin and Hygromycin and then verified by DNA sequencing. The strain whose *kstD1* was deleted is marked as Δ*kstD1*.

### Overexpression of KstD1, KstD2 and KstD3 in Δ*kstD*1

pMV306 without promoter and with promoter (pMV306hsp) were used to construct plasmids for functional complementation of KstD1 and overexpression of KstD2 and KstD3 [39, 40]. Firstly, the *KstD* ORFs were cloned to the EcoRI and SalI sites of pMV306hsp. And the host promoter regions together with the ORFs of *KstDs* were amplified from *M. neoaurum* DSM 1381 genome and subcloned into pMV306 between BamHI and EcoRI sites. After being verified by DNA sequencing, the resulting pMV306hsp-*kstD1*/pMV306hsp-*kstD2*/pMV306hsp-*kstD3* and pMV306Pk1-*kstD1*/pMV306Pk2-*kstD2*/pMV306Pk3-*kstD3* were introduced in Δ*kstD1* by electroporation. The colonies of Δ*kstD1* harboring the right recombinant plasmids were picked and verified using the primers kan-f&r to check the presence of plasmids. The result recombinant strains were noted as HK1, HK2, HK3, PK1, PK2 and PK3, respectively. Then bioconversion of phytosterols using the recombinant Δ*kstD1* strains was performed to study the characteristics of the three KstD isozymes and their promoters in Δ*kstD1*.

### KstD enzymatic assay

Induced with 1 mM IPTG for 24 h, the cell pellets obtained at 6000 rpm for 10 min at 4 °C from 50 mL cultures of the recombination *E. coli* BL21 (DE3) and *B. subtili*s 6051a strains were resuspended in 4 mL 50 mM Tris–HCl buffer (pH 7.0) after twice washing and then sonicated for 10 min under the protection of ice-water bath. Then the supernatant of cell extracts (12,000 rpm, 4 °C, 5 min) was used for enzyme activity assay. And for the *M. neoaurum* DSM 1381 and Δ*kstD1*, after induced with 5 g L^−1^ phytosterols (33–36 h), the cells were collected in the same way. The KstD enzymatic activities of soluble part of both the cultures and cell extracts obtained at 12,000 rpm for 30 min at 4 °C were measured spectrophotometrically at 600 nm (ε_600_ = 18.7 × 10^3^ cm^−1^ M^−1^) by Thermo Scientific Nano Drop 2000 at 30 °C [28]. The reaction mixture (1 mL) contained 50 mM Tris–HCl pH 7.0, 1.5 mM PMS, 0.12 mM DCPIP, cell extracts or supernatants of cultures, 500 μM AD/4HP and 5 g L^−1^ HP-β-CD. The substrates were previously dissolved in HP-β-CD. Three replicates were analyzed. The enzymatic activity of the specific substrate of each sample was calculated by subtracting the value of the activity of control (without any steroid). The total protein content in the supernatants of cell extracts and cultures were quantified by Bradford assay [41]. 1 U of enzyme activity is defined as the reduction of 1 µmol of DCPIP/min.

### SDS-polyacrylamide gel electrophoresis of proteins (SDS-PAGE)

The samples used for SDS-PAGE were mixed with 5 × SDS loading buffer (Shanghai Songon) with ratio 4:1 (v/v). The mixture was then boiled in water for 10 min, and centrifuged for 10 min at 12,000 rpm. The samples were run on a SDS-PAGE as described by Laemmli [42].

### Analytical methods

The fermentation experiments of *M. neoaurum* DSM 1381 were sampled every 12 or 24 h and three replications were used to measure steroids. The samples were extracted three times with an equal volume of ethyl acetate, and then the three extracts were mixed to analyze with thin layer chromatography (TLC), high-performance liquid chromatography (HPLC) and gas chromatography (GC). TLC was used as a qualitative approach to detect the steroids bioconversion products with ethyl acetate-hexane (6:4) as a developing solvent. Samples in ethyl acetate were re-dissolved in methanol after drying. HPLC with Agilent Extend-C18 column (4.6 × 250 mm; 40 °C) was used to determine the 3-ketosteroids with methanol/water (80:20, v/v) as the mobile phase at a flow rate of 1 mL min^−1^ and the wave length of ultraviolet detector was 254 nm. To quantify phytosterols having no ultraviolet absorption, the chromatographic method was performed on a Rtx-5 (30 m × 0.53 mm × 5.0 µm) using squalene (Sigma) as an internal reference standard. GC-2010Plus (Shimadzu, Japan) with a flame ionization detector was employed. The temperatures of inlet, column and flame-ionization detector were 320, 300 and 320 °C, respectively.

### Accession numbers

The *kstD1*, *kstD2*, and *kstD3* ORF sequences from *M. neoaurum* DSM 1381 have been deposited in the GenBank database with the Accession Numbers MG251735, MG251736, and MG251737, respectively.

## Results

### In silico analysis of the three putative KstD isoenzymes

*M. neoaurum* DSM 1381’s genome were sequenced and annotated as described in “Methods”. Three putative *kstD* ORFs (*orf04645*, *orf05164*, and *orf05167*) were identified, and the ORF sequences have been deposited in GenBank database. The genetic organizations of the three *kstD* ORFs are shown in Additional file 2: Fig. S1. *orf04645*, hereinafter referred to as *kstD1*, was located within a gene cluster associated with steroid degradation and was surrounded by *hsaF* (4-Hydroxy-2-ketovalerate aldolase) and *hsd4B* (3β-Hydroxysteroid dehydrogenase/Δ^5^-Δ^4^-isomerase). *kstD2* (*orf05167*) was adjacent to *kstD3* (*orf05164*) and *kshA* (3-Ketosteroid 9α-hydroxylase oxygenase subunit). According to FgenesB, *kstD1* and *kstD2* were regulated independently by their promoters, and *kstD3* was located on an operon. Further, *KstD* expression is thought to be regulated through the binding of helix-turn-helix transcriptional repressors (KstRs) [32, 33]. UGENE 1.27.0 identified a putative KstR1 binding site before the *kstD1* gene with a quality parameter score of 89.26%. Nevertheless, no KstR1 binding site was predicted before *kstD2* and *kstD3*, and none of the three *kstDs* were under the control of KstR2.

**Fig. 2:**
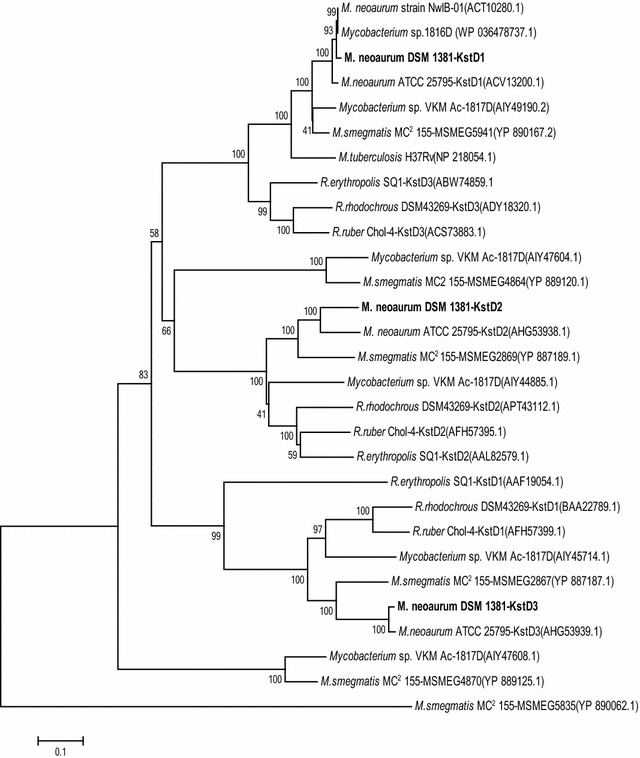
Phylogenetic tree of three KstDs in *M. neoaurum* DSM 1381 and their orthologues. 1000 replicates were set to generate bootstrap values when conducting the analysis

The nucleotide sequences of the *kstDs* were translated into aa sequences. A dendrogram was subsequently composed to elucidate evolutionary relationships between the three putative KstD proteins and their homologs, obtained from recent literature. As shown in Fig. 2, the aa sequence of KstD1 of *M. neoaurum* DSM 1381 shared a high identity with KstD1 of *M. neoaurum* ATCC 25795 (97%) [10], KstD1 of *Mycobacterium* sp. VKM Ac-1816D (99%) [25], KstD3 of *R. ruber* Chol-4 (66%) [28], and KstD3 of *R. erythropolis* SQ1 (67%) [31]. KstD3 of *M. neoaurum* DSM 1381 showed 97, 68, and 46% identity with the homologous protein of *M. neoaurum* ATCC 25795, *R. ruber* Chol-4 and *R. erythropolis* SQ1, respectively [10, 28, 29]. Unexpectedly, KstD2 of *M. neoaurum* DSM 1381 shared only 85% aa identity with the KstD2 of *M. neoaurum* ATCC 25795, but 68% and 65% aa identity with the KstD2 of *R. ruber* Chol-4 and *R. erythropolis* SQ1, respectively [10, 28, 30]. Moreover, through NCBI BLAST, the highest identity found that corresponds to the KstD2 sequence of *M. neoaurum* DSM 1381 was 85%, suggesting a new unidentified KstD.

### Heterologous expression of KstD1, KstD2 and KstD3 of *M. neoaurum* DSM 1381 in *E. coli* BL21 (DE3) and *B. subtilis* 6051a

The expression of KstDs in *E. coli* BL21 (DE3) or *B. subtilis* 6051a were identified through SDS-PAGE (Additional file 3: Fig. S2). To detect the activities of the KstDs, the biochemical properties of recombinant KstD1, KstD2, and KstD3 from *E. coli* BL21 (DE3) or *B. subtilis* 6051a were investigated (Table 2). Host cells with the blank plasmid were used as controls. The intracellular and extracellular KstD activities of BL21-*kstD1/*BL21-*kstD2/*BL21-*kstD3* and 6051a-*kstD1/*6051a-*kstD2/*6051a-*kstD3* on AD and 4HP were separately measured. As shown in Table 2, the KstD activity of BL21-*kstD2*, with AD and 4HP as substrates, were 3.46 and 2.82 U mg^−1^, respectively. Unexpectedly, recombinant KstD1 and KstD3 from *E. coli* BL21 (DE3) showed very low level of activities (≤ 0.34 U mg^−1^) on both AD and 4HP. However, the activities of intracellular soluble parts from 6051a-*kstD1* on AD and 4HP reached 2.12 and 1.81 U mg^−1^, respectively. The improvement on enzymatic activity of recombinant KstD1 was probably caused by poorly expressed enzyme *E. coli* due to the recombinant KstDs forming inclusion body in *E. coli,* as shown in Additional file 3: Fig. S2, which were also reported previously [21, 23]. And the same phenomenon was also noticed on the KstD2. The intracellular KstD activity of recombinant 6051a-*kstD2* reached 22.40 and 19.19 U mg^−1^, respectively. Nevertheless, the recombinant KstD3’s activity was still negligible in *B. subtilis,* further verifying the poor activity of KstD3.

**Table 2:** The KstD enzyme activity (U mg^−1^ total soluble protoplast protein and extracellular protein) of *M. neoaurum* DSM 1381, Δ*kstD1*, recombinant *E. coli* BL21 (DE3), and *B. subtilis* 6051a strains

Strains	Substrate			
	AD		4HP	
	Intracellular enzyme	Extracellular enzyme	Intracellular enzyme	Extracellular enzyme
DSM 1381	2.45 ± 0.05	u.d.	2.00 ± 0.19	u.d.
Δ*kstD1*	0.14 ± 0.01	u.d.	0.06 ± 0.01	u.d.
6051a-pHT01	u.d.	u.d.	u.d.	u.d.
6051a-*kstD1*	2.12 ± 0.06	u.d.	1.81 ± 0.05	u.d.
6051a-*kstD2*	22.40 ± 1.26	u.d.	19.19 ± 0.96	u.d.
6051a-*kstD3*	0.18 ± 0.06	u.d.	0.15 ± 0.06	u.d.
BL21-pET28a	u.d.	u.d.	u.d.	u.d.
BL21-*kstD1*	0.34 ± 0.04	u.d.	0.21 ± 0.05	u.d.
BL21-*kstD2*	3.46 ± 0.07	u.d.	2.82 ± 0.12	u.d.
BL21-*kstD3*	0.29 ± 0.08	u.d.	0.32 ± 0.14	u.d.

All assays were performed with triplicate cultures. Standard deviations of the biological replicates are shown

DSM1381, *M. neoaurum* DSM 1381; Δ*kstD1*, mutant of *M. neoaurum* DSM 1381 deleted kstD1; BL21-*kstD1/*BL21-*kstD2/*BL21-*kstD3*, *E. coli* BL21 (DE3) containing pET28a-*kstD1/*pET28a-*kstD2/*pET28a-*kstD3*; BL21-pET28a, *E. coli* BL21 (DE3) containing empty plasmid pET28a(+); 6051a-*kstD1/*6051a-*kstD2/*6051a-*kstD3*, *B. subtilis* 6051a containing pHT01-*kstD1/*pHT01-*kstD2/*pHT01-*kstD3*; 6051a-pHT01, *B. subtilis* 6051a containing empty plasmid pHT01

*u.d.* undetectable enzyme activity

In conclusion, among the three KstDs, KstD2 showed the highest enzymatic activity when expressed heterogeneously, and KstD1 performed poorly, especially in *E. coli*. In addition, for KstD3, the KstD enzyme activities were hardly detected in either host.

### Bioconversion of AD and 4HP by the recombinant *E. coli* BL21 (DE3) and *B. subtilis* 6051a

**Fig. 3:**
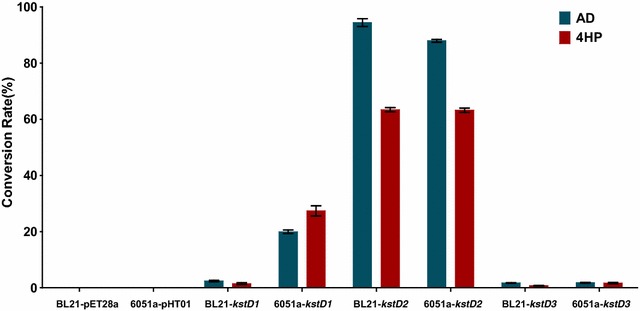
AD and 4HP bioconversion assays of recombinant strains. LB medium supplied with 1 g L^−1^ AD or 4HP was used to test the transformation ability of the recombinant *E. coli* BL21 (DE3) and *B. subtilis* 6051a strains. BL21-*kstD1/*BL21-*kstD2/*BL21-*kstD3*, *E. coli* BL21 (DE3) containing pET28a-*kstD1/*pET28a-*kstD2/*pET28a-*kstD3*; BL21-pET28a, *E. coli* BL21 (DE3) containing empty plasmid pET28a(+); 6051a-*kstD1/*6051a-*kstD2/*6051a-*kstD3*, *B. subtilis* 6051A containing pHT01-*kstD1/*pHT01-*kstD2/*pHT01-*kstD3*; 6051a-pHT01, *B. subtilis* 6051a containing empty plasmid pHT01

**Fig. 4:**
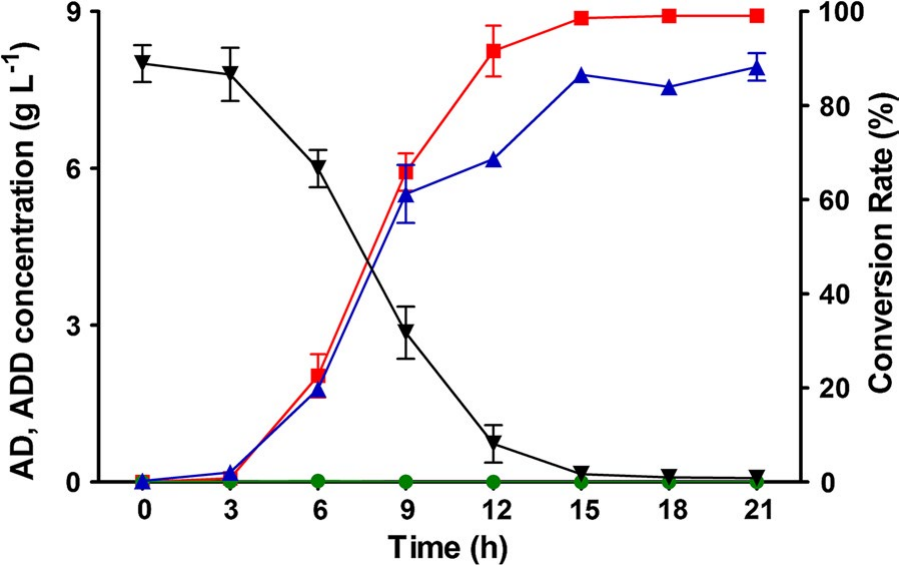
Time course of ADD accumulation from AD by BL21-*kstD2* and BL21- pET28a. BL21-*kstD2*, *E. coli* BL21 (DE3) containing pET28a-*kstD2*; BL21-pET28a, *E. coli* BL21 (DE3) containing empty pET28a(+); Green circle, conversion rate by BL21-pET28a; black inverse triangle, concentration of remained AD by BL21-*kstD2*; blue triangle, real-time yield of ADD by BL21-*kstD2*; red square, conversion rate by BL21-*kstD2*. Standard deviations of the biological replicates are represented by error bars

The gene expression and activity of the three KstDs of *M. neoaurum* DSM 1381 in *E. coli* and *B. subtilis* was tested in shake flask fermentation process (Fig. 3). Considering the low solubility of steroids in purified water, both HP-β-CD and Tween 80 were used to improve AD and 4HP solubility in the fermentation medium. The KstDs displayed different AD and 4HP conversion capacities in the different hosts. After 12 h of fermentation, 6051a-*kstD1* transformed 20.48% of AD to ADD and 27.41% of 4HP to HPD. By comparison, BL21-*kstD1* performed poorly. These observations were consistent with the KstD enzyme activity assay results. Further, KstD3 was only able to transform a maximum of 2.39% substrate in either of the host. Remarkably, BL21-*kstD2* converted as much as 95% of AD and 63.41% of 4HP in 12 h. Even though 6051a-*kstD2* was more active than BL21-*kstD2*, based on the enzyme activity assay, KstD2 in recombinant *B. subtilis* was only able to convert 88.52% of AD to ADD and 63.31% of 4HP to HPD. One of the reasons for the lower conversion rate with 6051a-*kstD2* was that the KstD activity of BL21-*kstD2* is higher than that of 6051a-*kstD2* during the early stage after the IPTG induction, prior to the formation of inclusion bodies. The steroid conversion results further demonstrated that KstD2 displays a high dehydrogenation activity and that BL21-*kstD2* is an excellent ADD producer. To maximize the AD dehydrogenation capacity of BL21-*kstD2*, AD fermentation was performed in Terrific Broth medium. Under the condition described in “Methods”, BL21-*kstD2* was capable of transforming 8 g L^−1^ of AD to ADD at a 99% conversion rate in 15 h (Fig. 4).

### Transcriptional analysis of *kstD* genes in *M. neoaurum* DSM 1381

**Fig. 5:**
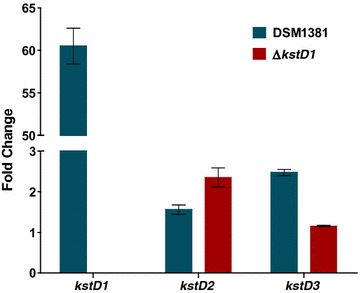
Analysis of *kstD* genes transcription in *M. neoaurum* DSM 1381 and Δ*kstD1* strains. The fold change indicates the ratio of phytosterols supplemented to control culture mRNA levels for the indicated strains. Data were calculated from three independent experiments and standard deviations are shown

The transcription levels of the three *kstD* genes in *M. neoaurum* DSM 1381 were analyzed by RT-qPCR following an addition of phytosterols. Cultures grown in MP01 medium containing 5 g L^−1^ phytosterols and control cultures grown in same medium without added steroids were collected to extract total RNA. As shown in Fig. 5, *kstD1* transcripts increased 60.5-fold in *M. neoaurum* DSM 1381 when induced with phytosterols. However, only a 1.6- and 2.5-fold increase in, respectively, *kstD2* and *kstD3* transcription was observed in response to the same treatment. These results were consistent with the gene sequencing analysis where a putative KstR binding site was only identified before *kstD1*. Taken together, only *kstD1* in *M. neoaurum* DSM 1381 was believed to be involved in the transformation of phytosterols. Further, the genetic regulation of *kstD* homologs, belonging to the same phylogenetic tree branch, seemed to be conserved. For example, a 4.5-, 13-, and 240.5-fold upregulation of, respectively, *kstD1* of *M. neoaurum* ATCC 25795 [10], *MSMEG_5941* of *M. smegmatis* mc^2^155 [13], and *kstD3* of *R. ruber* Chol-4 [28] were observed following an induction with cholesterol. Moreover, in *M. neoaurum* ATCC 25795, only *kstD1* and *kstD3* contributed to the accumulation of ADD from cholesterol. After deleting *MSMEG_5941*, a homolog of *M. neoaurum* DSM 1381 *kstD1*, the AD molar yield for phytosterols became 84%, and only a small amount of ADD and HPD were accumulated in ΔkshB1 *M. smegmati* mutant [13]. This may suggest that among the three *kstDs*, only *kstD1* was involved in the transformation of phytosterols, which would agree with earlier reports for other *Mycobacterium* strains. Based on our results it was anticipated that the deletion of the *kstD1* gene would inhibit the conversion of 4HP to HPD.

### Construction of the Δ*kstD1 M. neoaurum* DSM 1381 mutant

As previously reported, the Δ*kstD1 M. neoaurum* DSM 1381 mutant clones that lost their *hyg* resistance were verified by PCR and the DNA fragment of the desirable mutants was sequenced [36], from which it was found that only one *dif* site was left between the upstreasm and downstream of *kstD1*. Then, the phenotypes of the Δ*kstD1* mutant were studied, and its transcription levels of *kstD2* and *kstD3* were quantified. Following the deletion of KstD1, the expressions of *kstD2* and *kstD3* rose 2.4- and 1.4-fold, respectively (Fig. 5). This strongly suggested that even if *kstD1* was deleted, *kstD2* and *kstD3* could not be induced by phytosterols. Further, compared to the wild-type, the intracellular enzyme activity of Δ*kstD1* on AD had decreased from 2.45 to 0.14 U mg^−1^ (Table 2). Also, Δ*kstD1* showed only slight activity for 4HP (Additional file 4: Fig. S3). Finally, when fermented with 5 g L^−1^ phytosterols, the *M. neoaurum* DSM 1381 wild-type produced 0.2 g L^−1^ 4HP, 3.0 g L^−1^ HPD, 17.8 mg L^−1^ AD, and 32.5 mg L^−1^ ADD over 132 h, while the Δ*kstD1* accumulated mainly 4HP (2.8 g L^−1^). Yields of HPD, AD, and ADD were merely 0.1 g L^−1^, 48.4 mg L^−1^, and 9.4 mg L^−1^, respectively. Overall, *kstD1* has been shown to play a major role in the production of HPD from 4HP. As expected, the Δ*kstD1* mutant mainly produced 4HP.

### *kstD1*, *kstD2* and *kstD3* expression affects the molar ratio of HPD/4HP and ADD/AD in Δ*kstD1*

**Fig. 6:**
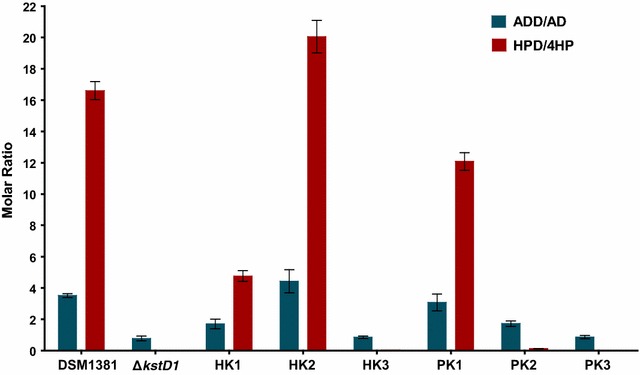
Molar ratio of ADD/AD and HPD/4HP of *M. neoaurum* DSM 1381 and its recombinant strains. The assay was carried out with MP01 medium supplied with 5 g L^−1^ phytosterols. DSM1381, *M. neoaurum* DSM 1381; Δ*kstD1*, mutant of *M. neoaurum* DSM 1381 deleted *kstD1*; HK1/HK2/HK3, Δ*kstD1* harboring pMV306hsp-*kstD1*/pMV306hsp-*kstD2*/pMV306hsp-*kstD3*; PK1/PK2/PK3, Δ*kstD1* harboring *kstD1/kstD2/kstD3* expression plasmids under control of their own promoters

The KstDs were overexpressed in the Δ*kstD1* mutant, either using a strong constitutive promoter (*hsp60*), or under the control of their native promoter, to determine their specific function in the transformation of phytosterols to HPD. The molar yields of AD, ADD, 4HP and HPD after 144 h fermentation with 5 g L^−1^ phytosterols were measured (Table 3) and compared to the wild type *M. neoaurum* DSM 1381 (Fig. 6).

**Table 3:** Comparison of the molar yields (%) of products of Δ*kstD1* and its recombinant strains using 5 g L^−1^ phytosterols

Strains	DSM 1381	Δ*kstD1*	HK1	HK2	HK3	PK1	PK2	PK3
ADD	0.99 ± 0.11	0.62 ± 0.17	1.01 ± 0.06	1.07 ± 0.27	0.49 ± 0.08	0.94 ± 0.16	1.38 ± 0.34	0.59 ± 0.22
AD	0.28 ± 0.04	0.8 ± 0.15	0.61 ± 0.15	0.3 ± 0.06	0.57 ± 0.09	0.31 ± 0	0.8 ± 0.22	0.52 ± 0.12
HPD	68.79 ± 6.76	1.63 ± 0.13	59.37 ± 5.62	72.19 ± 2.03	2.22 ± 0.21	67.96 ± 3.9	8.84 ± 0.26	1.8 ± 0.31
4HP	3.8 ± 0.63	67.01 ± 0.37	12.52 ± 1.68	3.47 ± 0.54	68.07 ± 1.55	5.63 ± 0.4	66.42 ± 2.72	68.82 ± 2.94

DSM1381, *M. neoaurum* DSM 1381; Δ*kstD1*, mutant of *M. neoaurum* DSM 1381 deleted kstD1; HK1/HK2/HK3, Δ*kstD1* harboring pMV306hsp-*kstD1*/pMV306hsp-*kstD2*/pMV306hsp-*kstD3*; PK1/PK2/PK3, Δ*kstD1* harboring *kstD1*/*kstD2*/*kstD3* expression plasmids under control of their own promoter

Data for the constitutively expressed KstDs showed that KstD2 has a higher Δ1-dehydrogenation activity than KstD1 since the HPD/4HP molar ratio of HK2 (20.05:1) was much higher than HK1 (4.76:1) and even higher than *M. neoaurum* DSM 1381 (16.61:1). Further, the HPD/4HP molar ratios of HK3 (0.03:1) only slightly exceeded those of the Δ*kstD1* mutant (0.02:1), implying that the Δ1-dehydrogenation activity of KstD3 is negligible. The HPD/4HP molar ratio of PK1, or in other words the native expression of KstD1 in Δ*kstD1*, could be recovered to 12.09:1. And it is worth noting that the HPD molar yield of PK2 was 8.84%, much higher than in Δ*kstD1* (1.63%), and this might be due to the use of a weak native promoter and the concomitant high activity of KstD2, based on our RT-qPCR results and heterologous expression experiments. It is not thought that this lack of KstD3 activity in PK3 was related to the native promoter of KstD3 in the cassette used for KstD3 overexpression, as the KstD3 activity on 4HP and AD was also negligible in HK3. In conclusion, *kstD1* had a strong promoter with high Δ1-dehydrogenation activity in *M. neoaurum* DSM 1381; *kstD2* was found to play only a minor role in the phytosterols conversion; and the effect of *kstD3* appeared negligible.

### Phytosterols conversion capacity of the Δ*kstD1* mutant

**Fig. 7:**
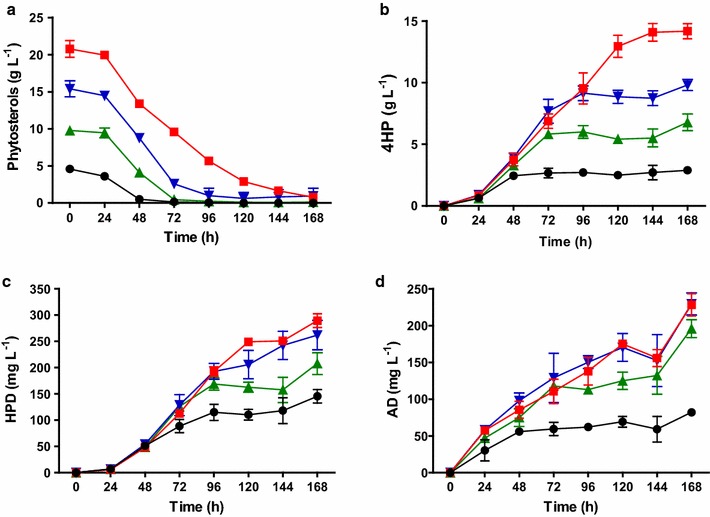
Time course of products accumulation from phytosterols by Δ*kstD1.* 1.5 parts of HP-β-CD to 1 part phytosterols was used to enhance bioconversion. Black circle, 5 g L^−1^ phytosterols; green triangle, 10 g L^−1^ phytosterols; blue inverse triangle, 15 g L^−1^ phytosterols; red square, 20 g L^−1^ phytosterols. HP-β-CD with 1.5:1 ratio to phytosterols was employed to enhance the bioconversion. **a** The concentration of remained phytosterols. **b**–**d** Real-time yield of 4HP, HPD and AD, respectively. All assays were performed in triplicate. Standard deviations of the biological replicates are represented by error bars

Figure 7 showed that the conversion rates of 5, 10, 15 and 20 g L^−1^ phytosterols over 168 h of fermentation was 100, 98.7, 98.6, and 96.3%, respectively. Specifically, in the space of 96 h, Δ*kstD1* was shown to have transformed all 5 g L^−1^ phytosterols into 2.88 g L^−1^ 4HP, 0.15 g L^−1^ HPD, and 82.10 mg L^−1^ AD. The yield of 4HP increased to 6.78, 9.80, and 14.18 g L^−1^ when fed with 10, 15, and 20 g L^−1^ phytosterols, respectively. The purities of 4HP were 92.3, 94, 95, and 96.12%, respectively, and this was thought to be the result of a continuous conversion from 4HP to HPD after most of the substrates were utilized. The former was deduced from our observations showing a similar 4HP/HPD molar ratio for 5 g L^−1^ phytosterols after 48 h (47.65:1) as for 20 g L^−1^ phytosterols after 168 h (48.99:1); and the latter being higher than the 4HP/HPD ratio for 5 g L^−1^ phytosterols after 168 h (18.5:1). Thus, elongation of the fermentation time would decrease the purity of 4HP but would benefit the conversion rate. This may have been due to the native expression of other KstDs and the pathway accumulating AD.

## Discussion

Many mutants have already been obtained using conventional breeding methods to produce a variety of drug intermediates and raw materials [1, 43]. However, the underlying mechanisms have only been studied for the last 20 years [3, 44, 45]. As whole-genome sequencing and transcriptome sequencing becomes more affordable, there is an increasing interest to identify the genes encoding key enzymes in actinobacteria [10, 25]. In fact, many mutants, such as *M. neoaurum* DSM 1381 [11] and *Mycobacterium* sp. VKM Ac-1815D [25], already have a valuable gene database that can be used as a tool for constructing novel strains to meet the demands of the pharmaceutical industry. *M. neoaurum* DSM 1381, a HPD/4HP producing *M. neoaurum* mutant [11], was considered a good candidate strain to explore the steroid degradation pathway responsible for the accumulation of HPD. To the best of our knowledge, this paper is the first time to present the performance of three KstDs isoenzymes during phytosterols conversion in *M. neoaurum* DSM 1381.

**Fig. 8:**
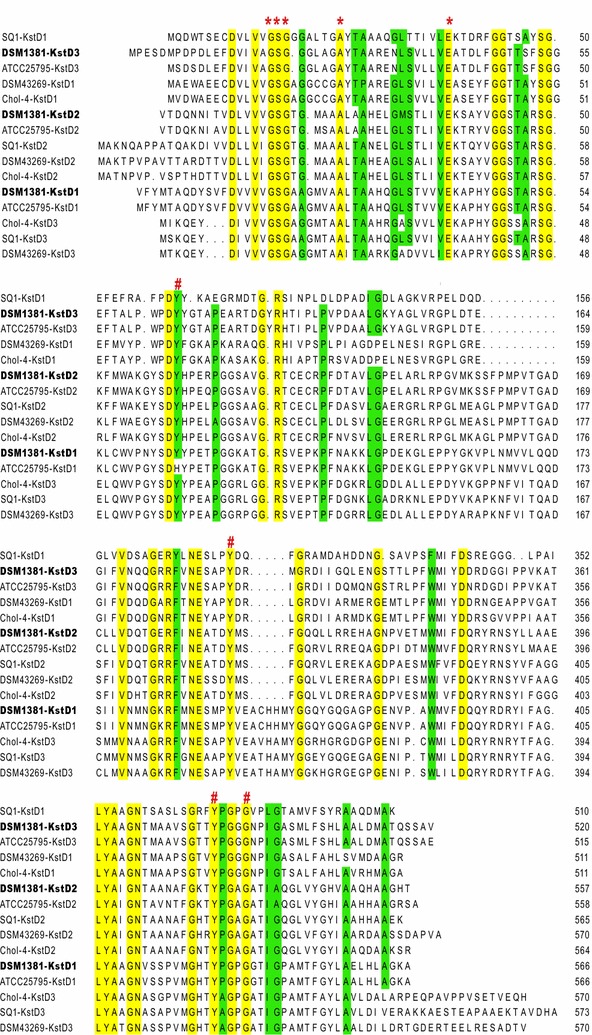
The sequence alignment of known KstD enzymes. DSM1381-KstDs from *M. neoaurum* DSM 1381, ATCC25795-KstDs from *M. neoaurum* ATCC 25795, DSM43269-KstDs from *R. rhodochrous* DSM 43269, Cho1-4-KstDs from *R. ruber* strain Chol-4, SQ1-KstDs from *R. erythropolis* SQ1. Active site residues and residues involved in co-ordination of a FAD in SQ1-KstD1 are indicated by number sign. A conserved sequence for FAD-binding region is indicated by asterisk

In this study, three *kstD* genes were identified in the *M. neoaurum* DSM 1381 genome. It is worth noting that, even though *M. neoaurum* DSM 1381 and *M. neoaurum* ATCC 25795 belong to the same species, the aa sequences of the KstDs in this study have highlighted certain differences. KstD1 shared a high aa sequence identity with its homologs of other *M. neoaurum* strains. For the KstD3, the shared aa identity of *M. neoaurum* ATCC 25795 and *M. neoaurum* DSM 1381 was as high as for KstD1 (97%). KstD2, on the other hand, only shared 85% aa identity with KstD2 from *M. neoaurum* ATCC 25795 [10] which was concomitantly also the highest shared aa identity following a blast search. KstD2 was therefore considered a new enzyme, different from previously reported KstDs. As shown in Fig. 8, the three isoenzymes are flavoproteins containing a consensus N-terminal flavin adenine dinucleotide (FAD)-dependent domain (GSGX_5–6_AX_2_AX_8_E) [10, 19]. Four residues, considered significant for flavoprotein functioning, were found to be highly conserved in the three KstDs: Tyr^119^, Tyr^487^, and Gly^491^, in the FAD-binding domain, and Tyr^318^ in the catalytic domain [45]. Nevertheless, mutations of other sites of KstD enzymes also have been shown to influence their activity. For example, p.S138L decreased the activity of KstD1 of *M. neoaurum*, whereas a p.V366S increased its activity [46, 47]. Furthermore, a p.Y125H substitution of the KstD1 from *M. neoaurum* ATCC 25795 had only a relatively small effect [10]. We would further study the function of the three isoenzymes to further our understanding and elucidate the reaction mechanisms of the KstD enzymes.

According to our results, the performance of KstD1 from *M. neoaurum* DSM 1381 is much better in *B. subtili*s than that in *E. coli*. This is in agreement with the performance of its homolog from *M. neoaurum* JC-12 and implied that these two KstDs probably shared the same characteristics [21]. Compared with the KstD3 from *M. neoaurum* ATCC 25795, the KstD3 from *M. neoaurum* DSM 1381 hardly showed detectable activity on both AD and 4HP [10]. Following the analysis of its aa sequence, it is possible that these observations can be attributed to the eight substitutions near Tyr^318^ in the catalytic domain of KstD3. KstD2 of *M. neoaurum* DSM 1381 showed remarkably high activities on both AD and 4HP. The properties of KstD2 offer exciting prospects to increasing our knowledge of the catalytic mechanisms of KstDs and as an application for drug development. The latter is owing to the ease by which KstD2 can be expressed in both commonly used hosts, whereas most other well-studied KstDs can only be actively expressed at low temperature or by addition of osmolytes [19, 23, 28]. Taken together, the research conducted so far suggests that KstD2 of *M. neoaurum* DSM 1381 has several characteristics that would favor its use on an industrial scale.

Recently, several KstDs were expressed in common hosts to construct industrial stains [23]. In *E. coli*, 6 g L^−1^ hydrocortisone can currently be transformed into prednisolone when expressing MsKstD1 from *M. smegmatis* mc^2^155 [19]. *P. pastoris* expresssion KstD_F_ from *Aspergillus fumigatus* CICC 40167 is able to transform 1 g L^−1^ AD to ADD after 4 h of fermentation, however, the cell culture needs a further 6 days to establish [20]. In addition, KstD1 from *M. neoaurum* JC-12 has been expressed in *C. crenatum*, *E. coli* and *B. subtili*s to produce ADD from AD [22–24]. The highest yield (8.76 g L^−1^) was obtained in *B. subtili*s, with a fed-batch strategy in 50 h, after codon optimization and co-expression with catalase to remove H_2_O_2_ toxicity [24]. Most of the research was carried out using a whole-cell biocatalyst method. The optimum temperature of these KstDs is 30 or 25 °C, which does not promote bacterial growth [19, 22]. Nonetheless, these KstD activities are still quite low. The discovery of KstD2 of *M. neoaurum* DSM 1381 is therefore of great importance. Compared to previously studied KstDs, the BL21-*kstD2* clone, expressing KstD2 of *M. neoaurum* DSM 1381, effectively transformed up to 8 g L^−1^ AD to ADD after 15 h of fermentation. In brief, BL21-*kstD2* is believed to be a promising industrial strain for the effective transformation of 4-ene-3-oxosteroids.

In *M. neoaurum* DSM 1381, most of the KstD activities were contributed by *kstD1* according to RT-qPCR and overexpression experiment results. In agreement with earlier reports of KstD1 homologues from *Rhodococcus* to *Mycobacterium*, kstD1 expression in *M. neoaurum* DSM 1381 was induced by phytosterols [13, 25, 28], a TetR-type transcriptional regulator involved in the steroid metabolism within actinobacteria, in the upstream region of *kstD1.* Compared to the *kstD2* homologs in *M. neoaurum* ATCC 25795, KstD2 of *M. neoaurum* DSM 1381 showed a much higher activity on AD but only played a minor role in phytosterols conversion to 4HP, possibly due to its weak gene expression levels [10]. Its homolog in *M. smegmatis* also showed little effect on AD degradation. However, KstD2 homologs in *R. rhodochrous* DSM43269 [26], *R. ruber* strain Chol-4 [27, 28] and *R. erythropolis* SQ1 [29–31] were the main contributors to AD degradation. The operon containing *kstD3* and controlled by a promoter could not be induced by phytosterols. The effect of KstD3 on phytosterols conversion to HPD is considered negligible since both its transcription and activity were low. As reported for *M. neoaurum* ATCC 25795, KstD3 played a role in AD metabolism but had no obvious impact on the Δ1-dehydrogenation of 4HP [10, 12]. Its homolog in *M. smegmatis* showed high activities on multiple substrates but made little contribution to AD degradation. *R. rhodochrous* DSM43269 [26], *R. ruber* strain Chol-4 [27, 28] and *R. erythropolis* SQ1 [31] harbor an active KstD3 homolog (Fig. 8).

Compared to rhodococci, mycobacteria are better hosts for construct 4HP or HPD producers, as the corresponding C22 intermediate of *Rhodococcus* is 4-pregnen-3-one-20β-carboxylic acid (4-BNC) rather than 4HP, and the reason for this has remained elusive to date [17]. In this paper, the molar yield of 4HP increased from 3.8 to 67.01%, after the deletion of *kstD1* in *M. neoaurum* DSM 1381, which is much higher than the XIIΔ*hsd4A*Δ*kstD123* mutant of *M. neoaurum* ATCC 25795 (47–49%) [12]. In *M. neoaurum* ATCC 25795, 1–2% HPD accumulated even after *kstD1*, *kstD2*, *kstD3* were removed and would suggest that there are other dehydrogenases that might contribute to the degradation of steroids [12]. Therefore, it was thought that Δ*kstD1* could not be optimized further by a simple deletion of *kstD2* to eliminate the appearance of HPD. Further research into the transformation mechanism of 4HP to HPD would be required to remove the small amount of AD (0.8% molar yield). Nevertheless, the Δ*kstD1* mutant of *M. neoaurum* DSM 1381 was shown to be an excellent strain due to its high molar yield of 4HP (65–73%), a high capacity to utilize substrate (96.3% of 20 g L^−1^ phytosterols) and the low accumulation of byproducts.

## Conclusions

In the transformation of phytosterols to HPD in *M. neoaurum* DSM 1381, KstD1 has been shown to play a dominant role, whereas KstD2 was only a minor contributor and KstD3 activity was negligible. KstD2, in particular, was shown to be a novel and strong candidate for industrial applications as it demonstrated high activity and could easily be expressed in *E. coli* and *B. subtilis*. And the recombinant BL21-*kstD2* was proven to be a promising ADD producer. In addition, our research has led to the construction of an excellent 4HP producing strain, obtained by deleting *kstD1* in *M. neoaurum* DSM 1381. This Δ*kstD1* mutant could produce 14.18 g L^−1^ 4HP from 20 g L^−1^ phytosterols after 168 h of fermentation.

## Additional files

**Additional file 1: Table S1.** Primers used in this work.

**Additional file 2: Fig. S1.** Schematic of the genetic organization of KstD genes in *M. neoaurum* DSM 1381.

**Additional file 3: Fig. S2.** SDS-PAGE analysis of KstDs expression in *E. coli* BL21 (DE3) and *B. subtilis* 6051a.

**Additional file 4: Fig. S3.** HPLC chromatogram comparison of the products from the transformation of 5 g L^−1^ of phytosterols at 30 °C by strains Δ*kstD1* and *M. neoaurum* DSM 1381.

## Abbreviations

AD: 4-Androstene-3,17-dione
ADD: 1,4-Androstadiene-3,17-dione
9-OHAD: 9α-Hydroxy-4-androstene-3,17-dione
4HP: 22-Hydroxy-23, 24-bisnorchola-4-en-3-one
HPD: 22-Hydroxy-23, 24-bisnorchola-1,4-dien-3-one
9-OHHP: 9,22-Dihydroxy-23,24-bisnorchol-4-ene-3-one
4-BNC: 4-Pregnen-3-one-20β-carboxylic acid
ORF: open reading frame
PMS: phenazine methosulphate
DCPIP: 2,6-Dichlorophenol-indophenol
KstD: 3-Ketosteroid-Δ1-dehydrogenase
FadA5: thiolase FadA5
CYP125: cytochrome P450 125
CHO: cholesterol oxidase
Hsd4A: 17β-Hydroxysteroid dehydrogenase and β-hydroxyacyl-CoA dehydrogenase
KSH: 3-Ketosteroid-9α-hydroxylases
*hsaF*: 4-Hydroxy-2-ketovalerate aldolase
*hsd4B*: 3β-Hydroxysteroid dehydrogenase/Δ^5^-Δ^4^-isomerase
*kshA*: 3-Ketosteroid 9α-hydroxylase oxygenase subunit
LB: Luria–Bertani
HPLC: high performance liquid chromatography
GC: gas chromatography
