# Supplementary material for: Identification, function, and application of 3-ketosteroid Δ1-dehydrogenase isozymes in Mycobacterium neoaurum DSM 1381 for the production of steroidic synthons
Source: Microb Cell Fact. 2018 May 18;17:77. doi: 10.1186/s12934-018-0916-9 (PMC5960168; doi:10.1186/s12934-018-0916-9)
Supplement: Supplementary file 1 — Additional file 1: Table S1. Primers used in this work. [file 12934_2018_916_MOESM1_ESM.pdf]

Table S1 Primers used in this study.

Primers	Sequence
pET28a-kd1f&r	cagcaaatgggtcgcggatccGTGTTCTACATGACTGCCCAGGA/ctcgagtgcggccgcaagcttGGCCTTTCCAGCGAGATGC
pET28a-kd2f&r	cagcaaatgggtcgcggatccGTGACCGATCAGAACAACATCACC/ctcgagtgcggccgcaagcttTCAGGTGTGTCCGGCGGC
pET28a-kd3f&r	cagcaaatgggtcgcggatccATGCCTGAATCAGACATGCCTG/ctcgagtgcggccgcaagcttTCATACCGCTGAGCTCTGTGTC
pHT01-kd1f&r	caattaaaggaggaaggatccGTGTTCTACATGACTGCCCAGGA/cattaggcgggctgcccccgggTCAGGCCTTTCCAGCGAGA
pHT01-kd2f&r	caattaaaggaggaaggatccGTGACCGATCAGAACAACATCACC/cattaggcgggctgcccgggTCAGGTGTGTCCGGCGGC
pHT01-kd3f&r	caattaaaggaggaaggatccATGCCTGAATCAGACATGCCTG/cattaggcgggctgccccgggTCATACCGCTGAGCTCTGTGTC
hyg*dif* -f	ttgtcgacggagctcgaattc***agtaccgataagctacattatgtcaact***GCTAAGGATTTTTTTATCTGAA
hyg*dif* -r	atgggtcgcggatccgaattc***agttgacataatgtagcttatcggatct***CTTTAAATCCAGATATCCACGG
kd1Uf&r	ctcgagtgcggccgcaagcttCTTCTCAGCCATACGTGGCTCC/tcgagctccgtcgacaagcttGTCCTGGGCAGTCATGTAGAACA
kd1Df&r	ttaaacaaaattatttctagaATCCGACCAACAAGCCGAACCCG/ggataacaattcccctctagaCCGGATCGGAATGCAGGGGATT
306k1f&r	gcggatccagctgcagaattcGTGTTCTACATGACTGCCCAG/acgctagttaactacgtcgacTCAGGCCTTTCCAGCGAG
306k2f&r	gcggatccagctgcagaattcGTGACCGATCAGAACAACAT/acgctagttaactacgtcgacTCAGGTGTGTCCGGCGGCAT
306k3f&r	gcggatccagctgcagaattcATGCCTGAATCAGACATGCCTGATC/acgctagttaactacgtcgacTCATACCGCTGAGCTCTGTGTCGCC
k1-f&r	aatctagatatccatggatccCGGGCAGATCATCGGCCA/acatcgataagcttcgaattcTCAGGCCTTTCCAGCGAGA
k2-f&r	atggatccagctgcagaattcGATGTTCTTGAATCCGGTCGG/tacgtcgacatcgataagcttTCAGGTGTGTCCGGCGGC
k3-f&r	atggatccagctgcagaattcACCCGCCCGGGCCTCAGT/tacgtcgacatcgataagcttTCATACCGCTGAGCTCTGTGTC
kan-f&r	ATGCCTCTTCCGACCATCAAG/GCTCTGCCAGTGTTACAACCA
RTkd1f&r	GTGCCCGCCTGGATGGTCTT/CGCCGTAGTAGCGGTCGTAG
RTkd2f&r	CGCACCTACCTTGAAGCACTCG/CGAACGGGCGACACTCACAG
RTkd3f&r	GGCGGCATCTTCGTCAACC/CAGAACGGCAATGTGGTGGAA
16SrRNAf&r	CCTATGTTGCCAGCGGGTTATGC/GCGATTACTAGCGACTCCGACTTCA

Restriction sites are marked with underline and *dif* sites are marked in bold italic
