# Supplementary figures and images for: Identification, function, and application of 3-ketosteroid Δ1-dehydrogenase isozymes in Mycobacterium neoaurum DSM 1381 for the production of steroidic synthons
Source: Microb Cell Fact. 2018 May 18;17:77. doi: 10.1186/s12934-018-0916-9 (PMC5960168; doi:10.1186/s12934-018-0916-9)

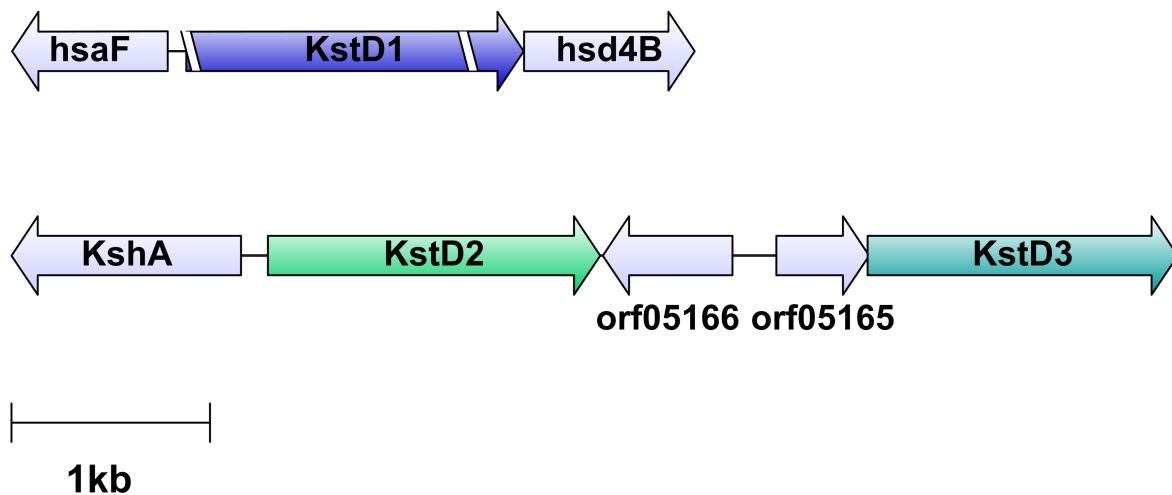

**Fig. S1** Schematic of the genetic organization of *kstD* genes in *M. neoaurum* DSM 1381

Supplement: Supplementary file 2 — Additional file 2: Fig. S1. Schematic of the genetic organization of KstD genes in M. neoaurum DSM 1381. [file 12934_2018_916_MOESM2_ESM.pdf]
