# Supplementary material for: Identification, function, and application of 3-ketosteroid Δ1-dehydrogenase isozymes in Mycobacterium neoaurum DSM 1381 for the production of steroidic synthons
Source: Microb Cell Fact. 2018 May 18;17:77. doi: 10.1186/s12934-018-0916-9 (PMC5960168; doi:10.1186/s12934-018-0916-9)
Supplement: Supplementary file 3 — Additional file 3: Fig. S2. SDS-PAGE analysis of KstDs expression in E. coli BL21 (DE3) and B. subtilis 6051a. [file 12934_2018_916_MOESM3_ESM.pdf]

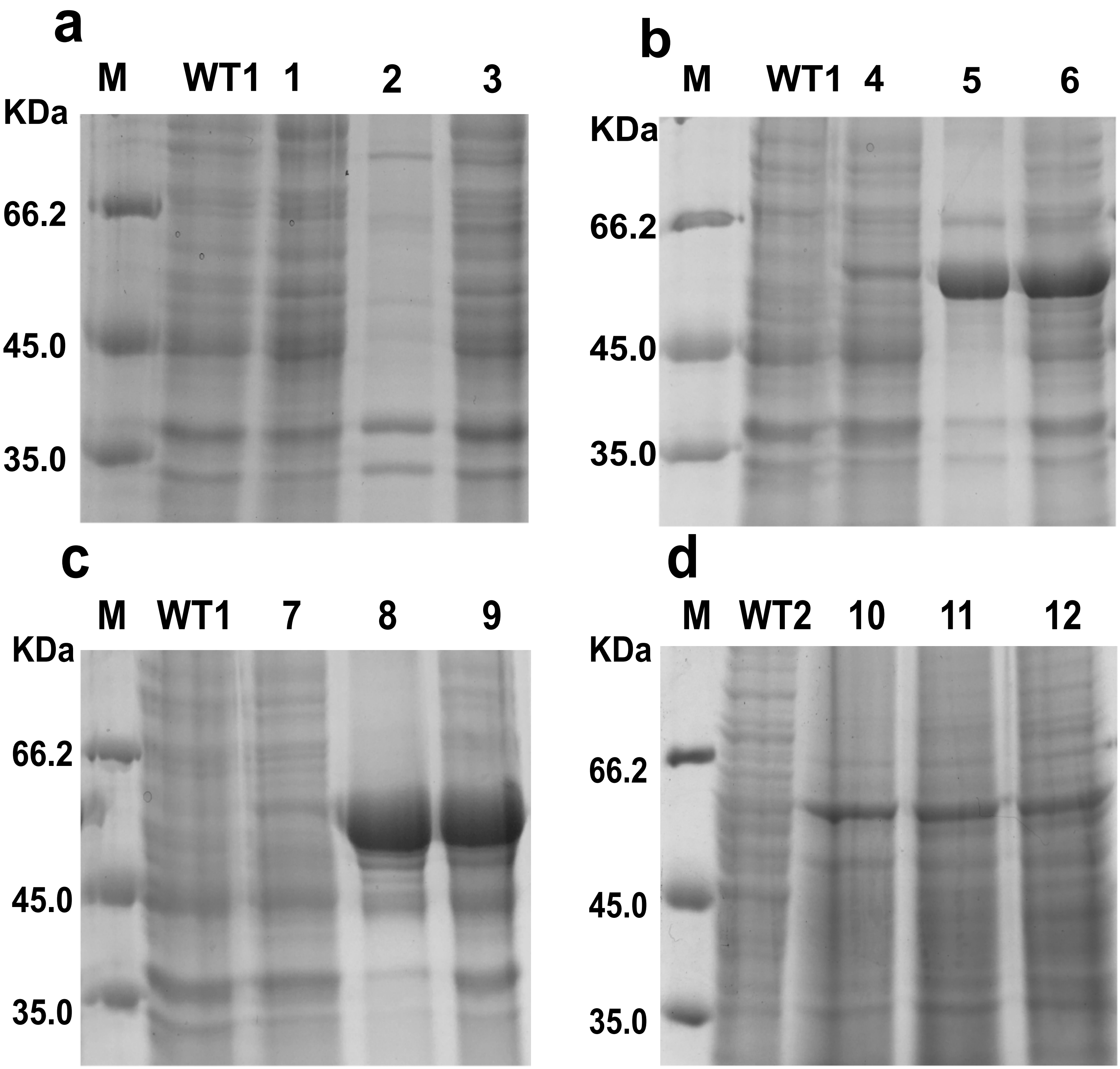

Fig. S2. SDS-PAGE analysis of expression of KstD1 (61.1 kDa), KstD2 (59.5 kDa), KstD3 (54.7 kDa) in *E. coli* BL21 (DE3) and *B. subtilis* 6051a.

Lanes: M, Protein markers; Lane WT1, total proteins in culture broth of BL21-pET-28a (+);
Lane 1, the supernatant of cell extracts of BL21-*kstD1*;
Lane 2, precipitation of cell extracts of BL21-*kstDS1*;
Lane 3, total proteins in culture broth of BL21-*kstD1*;
Lane 4, the supernatant of cell extracts of BL21-*kstD2*;
Lane 5, precipitation of cell extracts of BL21-*kstD2*;
Lane 6, total proteins in culture broth of BL21-*kstD2*;
Lane 7, the supernatant of cell extracts of BL21-*kstD3*;
Lane 8, precipitation of cell extracts of BL21-*kstD3*;
Lane 9, total proteins in culture broth of BL21-*kstD3*;
Lane WT2, the supernatant of cell extracts of 6051a-pHT01; Lane 10, the supernatant of cell extracts of 6051a-*kstD1*;
Lane 11, the supernatant of cell extracts of 6051a-*kstD2*; Lane 12, the supernatant of cell extracts of 6051a-*kstD3*.
