# Supplementary material for: Identification, function, and application of 3-ketosteroid Δ1-dehydrogenase isozymes in Mycobacterium neoaurum DSM 1381 for the production of steroidic synthons
Source: Microb Cell Fact. 2018 May 18;17:77. doi: 10.1186/s12934-018-0916-9 (PMC5960168; doi:10.1186/s12934-018-0916-9)
Supplement: Supplementary file 4 — Additional file 4: Fig. S3. HPLC chromatogram comparison of the products from the transformation of 5 g L−1 of phytosterols at 30 °C by strains ΔkstD1 and M. neoaurum DSM 1381. [file 12934_2018_916_MOESM4_ESM.pdf]

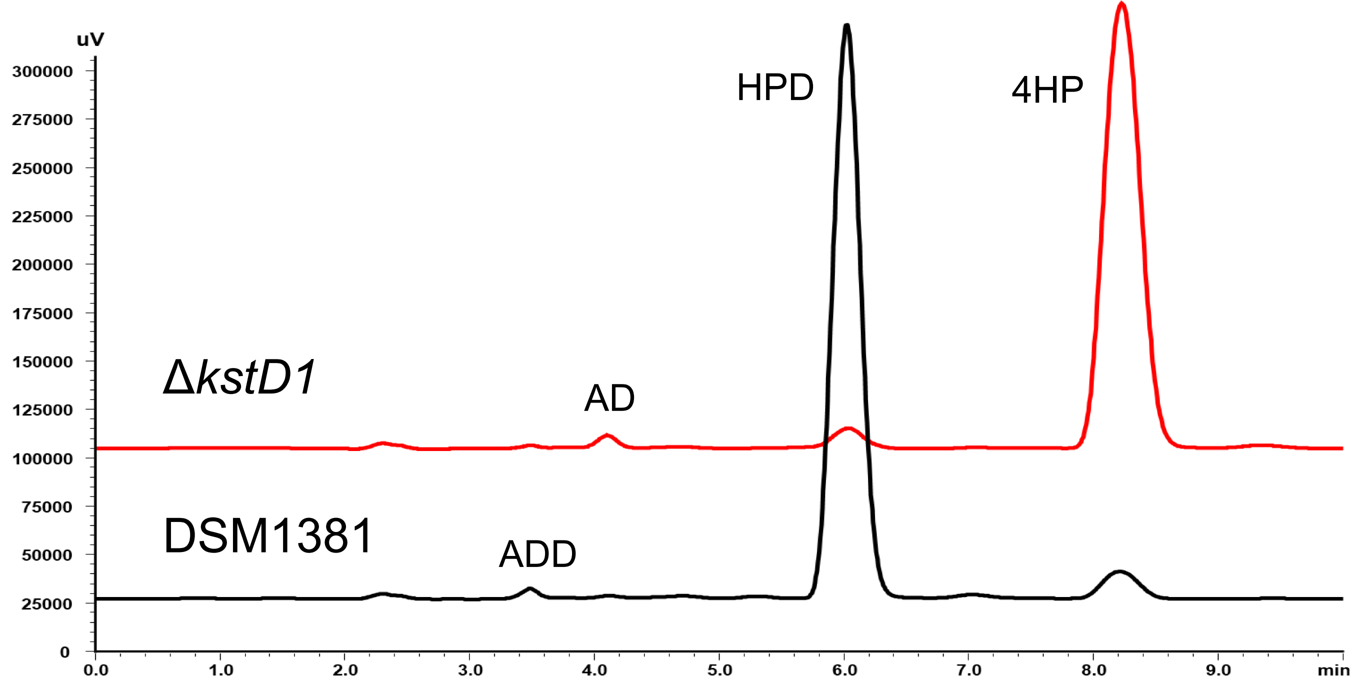

Fig. S3 HPLC chromatogram comparison of the products from the transformation of 5 g L^{-1} of phytosterols at 30 °C by strains Δ*kstD1* (red) and *M. neoaurum* DSM 1381 (black).
